# Effect of Blueberry Anthocyanins Malvidin and Glycosides on the Antioxidant Properties in Endothelial Cells

**DOI:** 10.1155/2016/1591803

**Published:** 2016-02-29

**Authors:** Wuyang Huang, Yunming Zhu, Chunyang Li, Zhongquan Sui, Weihong Min

**Affiliations:** ^1^Institute of Farm Product Processing, Jiangsu Academy of Agricultural Sciences, Nanjing 210014, China; ^2^College of Food Science and Engineering, Jilin Agricultural University, Changchun 130118, China; ^3^Department of Food Science and Engineering, School of Agriculture and Biology, Shanghai Jiao Tong University, Shanghai 200240, China

## Abstract

The objective of this research was to survey the antioxidant functional role of the main anthocyanins of blueberries in endothelial cells. Changes on the reactive oxygen species (ROS), xanthine oxidase-1 (XO-1), superoxide dismutase (SOD), and heme oxygenase-1 (HO-1) in cells of malvidin and the two glycosides were investigated. The results showed that these anthocyanins decreased the levels of ROS and XO-1 but increased the levels of SOD and HO-1. Glycosides improved the antioxidant capacity of malvidin to a great extent. The changes in the antioxidant properties of malvidin-3-glucoside were more pronounced than malvidin-3-galactoside. Variation in levels of malvidin-3-glucoside and malvidin-3-galactoside had a significant impact on antioxidant properties to different extents. It indicates that blueberries are a good resource of anthocyanins, which can protect cells from oxidative deterioration and use blueberry as a potential functional food to prevent diseases related to oxidative stress.

## 1. Introduction

There have been intense interest and active researches in the area of dietary antioxidants to develop functional food products [1]. Rabbiteye blueberries (*Vaccinium ashei*) are used as endogenous antioxidant defense ingredients in the modern health-conscious food industry, due to their significant levels of anthocyanins, phenolic acids, and vitamins [2, 3]. The health benefits of blueberries have been reported as reduction of coronary heart disease risk, visual improvement, and antimutagenic and anti-inflammatory effects [4]. The composition and molecular structure of anthocyanin determine the functional properties of blueberries [5]. Among fresh fruits and vegetables, blueberries contain greater amount of anthocyanin, especially malvidin-3-glucoside (Mv-3-glc) and malvidin-3-galactoside (Mv-3-gal) [6].

Malvidin possesses great antioxidant capacity with excellent free radical scavenging properties* in vitro* [7]. Malvidin exhibits antihypertensive activity by inhibiting angiotensin I-converting enzyme and anti-inflammatory effect by blocking NF-*κ*B pathway [8, 9]. Gopu et al. reported that malvidin interrupted quorum sensing in* Klebsiella pneumoniae* by docking with LasR receptor protein and reducing the violacein, EPS production, and biofilm formation [10]. In addition, malvidin plays a role in controlling both short- and long-term cellular activities. Several studies have shown that malvidin could inhibit different tumor cell lines* in vitro* or* in vivo*, including human promyelocytic/monocytic leukemia cells, gastric adenocarcinoma cells, and HT-29 colon cancer cells [11–13]. Matsunaga et al. found that malvidin could counteract oxidative stress in neuronal cells [14].

Malvidin presents in nature principally as the glycosylated form with the sugar moiety attached at position 3 on the c-ring, that is, malvidin-3-glucoside and malvidin-3-galactoside. Bioavailability of anthocyanins was extensively studied due to the different mechanisms of the uptake and metabolism (deconjugation, glucuronidation, sulfation, and deglucuronidation) in various cell lines. Passamonti et al. found Mv-3-glc possessed better bioavailability than other anthocyanidins due to the greater efficacy of binding to organic anion membrane carrier, bilitranslocase [15]. Rossetto et al. reported that Mv-3-glc showed synergistic antioxidant effect with catechin on free radical-initiated peroxidation of linoleic acid in micelles [16], and Grace et al. reported its significant hypoglycemic effect in diabetic C57b1/6J mice [17]. Additionally, Mv-3-glc is a potent anti-inflammatory agent* in vitro* and* in vivo*, without detectable toxicity on human peripheral blood mononuclear cells, which inhibits human macrophage-derived inflammatory mediators and decreases clinical scores in arthritic rats and inhibits ear oedema and leukocytes migration [18, 19]. Our previous study also found that Mv-3-glc and Mv-3-gal could inhibit TNF-*α*-induced inflammatory response in endothelial cells [20]. Quintieri et al. reported that Mv-3-glc could modulate mammalian myocardial and coronary performance and protect the heart against ischemia/reperfusion injury by activating the PI3K/NO/cGMP/PKG pathway and phosphorylating AKT and eNOS [21]. Paixão et al. confirmed the capacity of Mv-3-glc to increase NO bioavailability and to inhibit peroxynitrite-induced NF-*κ*B activation, supporting its benefits in cardiovascular health [22]. Anthocyanins could be as a promising tool for development of nutraceuticals to improve endothelial function. However, the antioxidant mechanisms of these anthocyanins in endothelial cells are still not clear. The objective of this study was to investigate the antioxidant functional role of the main anthocyanins in blueberries, malvidin, and its two glycosides (malvidin-3-glucoside and malvidin-3-galactoside) in human umbilical vein endothelial cells.

## 2. Materials and Methods

### 2.1. Chemicals and Reagents

Human umbilical vein endothelial cells (HUVECs) were obtained from Zhongqiaoxinzhou Biological Technology Co., Ltd. (Shanghai, China). Dulbecco's phosphate buffer saline, malvidin, malvidin-3-glucoside, malvidin-3-galactoside, and trypsin were obtained from Sigma Chemical Co., Ltd. (Nanjing, China). Fetal bovine serum and DMEM medium were obtained from Gibco/Invitrogen (Shanghai, China). Streptomycin and penicillin were obtained from Life Technologies (Shanghai, China). ROS Assay Kit was obtained from Biyotime Institute of Biotechnology (Shanghai, China). XO-1, SOD, and HO-1 ELISA Kit were obtained from Boster Biotechnology Inc. (Wuhan, China). All chemicals and reagents are of analytical grade.

### 2.2. Antibodies

Rabbit monoclonal primary antibody against XDH, mouse polyclonal primary antibodies to the *β*-actin, and goat anti-rabbit/mouse IgG-HRP conjugated secondary antibody were obtained from Boster Biotechnology Inc. (Wuhan, China). Primary antibodies against XDH were used at 1 : 400 dilutions, and primary antibodies against *β*-actin were used at 1 : 1000 dilutions. Secondary antibodies were used at 1 : 2000 dilutions.

### 2.3. Endothelial Cell Culture and Treatment

HUVECs could be characterized as a model system for studying oxidative stress in the vasculature. The cells were cultured according to a laboratory protocol [20]. After quiescing in a reduced serum medium for 4 h, the cells were treated with 1, 5, and 10 *μ*mol/L of Mv, Mv-glc, and Mv-gal, or mixture of Mv-glc and Mv-gal for 24 h, respectively. DMSO was instead in the control.

### 2.4. Reactive Oxygen Species (ROS) Assay

Dichloro-dihydro-fluorescein diacetate (DCFH-DA) assay is a quantitative method for oxidative stress assessment in cells. In this study, DCFH-DA detection kit was used to assess the ROS level in endothelial cells. Briefly, the cells were seeded in 6-well plates, treated with different samples to incubate for 24 h. After washing cells with PBS, 10 *μ*mol/L DCFH-DA was added to each well and reacted for 20 min at 37°C, and then the cells were washed thoroughly with PBS. A group of cells was visualized under an IX53 Inverted Fluorescent Microscope (Olympus, Tokyo, Japan) at 530 nm emission and 485 nm excitation filters immediately. All images presented are in ×200 magnification. Another one was collected in 1 mL PBS after dissociated, and fluorescence was recorded by LB 941 TriStar Microplate Reader (Berthold Technologies, Bad Wildbad, Germany). The total fluorescence intensity of cells in each well was noted, and ROS generation was measured as fold increase over the untreated control.

### 2.5. ELISA Analysis and Western Blotting

The levels of XO-1, SOD, and HO-1 in the supernatants were detected using ELISA kits according to the kit protocol booklet instructions. The absorbance was measured at 450 nm on a StatFax-2100 Microplate Reader (Awareness Technology Inc., Plam, FL, USA) via Hyper Terminal Applet ELISA software.

XO-1 protein expression was also analyzed by western blotting performed on the HUVEC lysates. *β*-actin was used as a loading control. All data were expressed as fold increase over the untreated control.

### 2.6. Statistical Analysis

Data were expressed as mean value ± standard deviation (SD) of triplicate determinations. Figures were obtained using GraphPad Prism Version 5 (GraphPad Software, Inc., CA, USA). One-way ANOVA was used for the determination of statistical significance using SPSS 19.0 Software. The differences were considered statistically significant with a *P* value of 0.05.

## 3. Results

### 3.1. Effects of Malvidin and Its Glycosides on Reactive Oxygen Species in Cells

**Figure 1:**
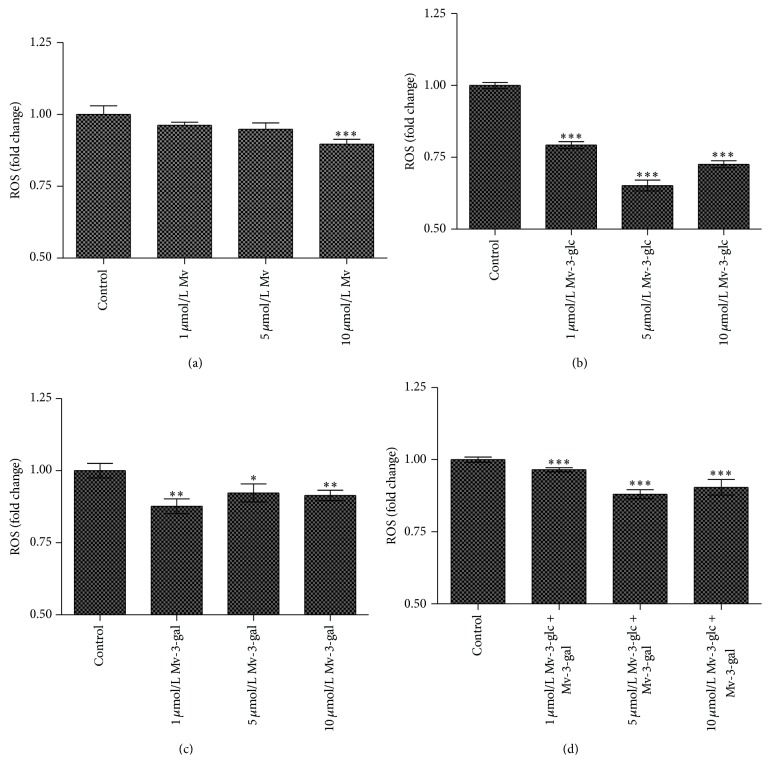
Effects of different concentrations Mv (a), Mv-3-glc (b), Mv-3-gal (c), and Mv-3-glc + Mv-3-gal (d) on the level of ROS in HUVECs. *∗*, *∗∗*, and *∗∗∗* indicate *P* < 0.05, *P* < 0.01, and *P* < 0.001, respectively, compared to the control.

**Figure 2:**
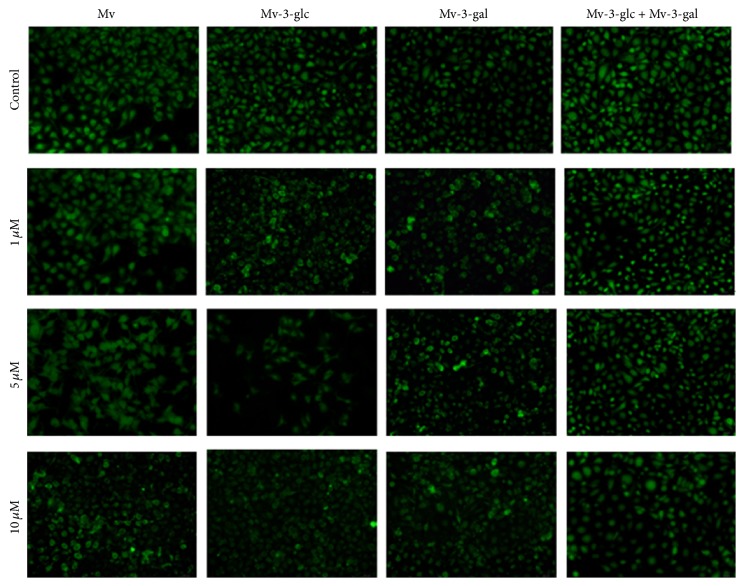
The fluorescence intensity of ROS in HUVECs treated with different concentrations Mv, Mv-3-glc, Mv-3-gal, and Mv-3-glc + Mv-3-gal. A representative set of images from three independent experiments is shown. All images presented are in ×200 magnification.

Addition of all of the tested malvidin, malvidin-3-glucoside, malvidin-3-galactoside, and the mixture of the two glycosides decreased ROS values in endothelial cells (Figure 1). Malvidin treated at 1, 5, and 10 *μ*mol/L concentration inhibited 4%, 6%, and 11% ROS, respectively. When Mv-3-glc was present, the values for ROS production decreased 21%, 35%, and 28% at the concentration of 1, 5, and 10 *μ*mol/L, respectively, whereas when Mv-3-gal was present, the values decreased 12%, 8%, and 9%, respectively. The mixture of Mv-3-glc and Mv-3-gal significantly decreased ROS level in cells. Both Mv-3-glc and the mixture of the two glycosides produced the greatest inhibition rate at the concentration of 5 *μ*mol/L, whereas Mv-3-gal produced the largest inhibition rate at the concentration of 1 *μ*mol/L. The extent of the decrease in ROS of Mv-3-glc was more pronounced than that of Mv-3-gal. However, there seemed to be an antagonism effect between Mv-3-glc and Mv-3-gal, since the mixture possessed lower ROS scavenging activity than Mv-3-glc. The fluorescence intensity levels of ROS showed similar effects (Figure 2).

### 3.2. Effects of Malvidin and Its Glycosides on XO-1 Production in Supernatant and Cells

**Figure 3:**
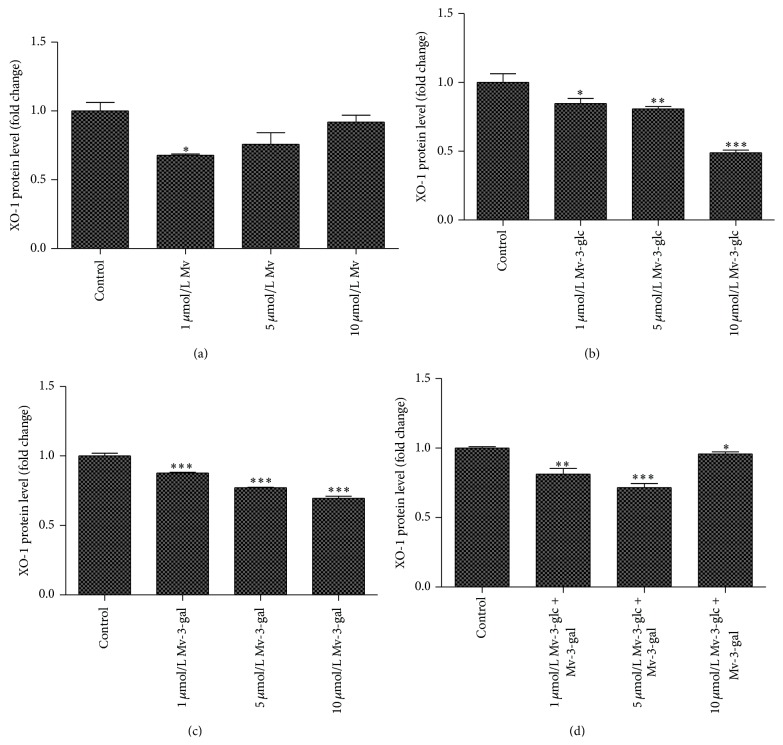
Effects of different concentrations Mv (a), Mv-3-glc (b), Mv-3-gal (c), and Mv-3-glc + Mv-3-gal (d) on XO-1 production released into the supernatant. *∗*, *∗∗*, and *∗∗∗* indicate *P* < 0.05, *P* < 0.01, and *P* < 0.001, respectively, compared to the control.

Addition of all the tested Mv, Mv-3-glc, Mv-3-gal, and the mixture decreased XO-1 production in supernatant (Figure 3). When the values of XO-1 production in supernatant treated with 1, 5, and 10 *μ*mol/L malvidin were compared to those of the control, they were found to be 0.68, 0.76, and 0.92 times, respectively. Interestingly, only Mv treated at lower concentration (1 *μ*mol/L) had significant inhibitory effect on XO-1 protein. The cells treated with 1, 5, and 10 *μ*mol/L Mv-3-glc, Mv-3-gal, and the mixture all significantly inhibited XO-1 level at the rate of 15%, 19%, 51%, and 13%, 23%, 31%, and 19%, 29%, 5%, respectively. The two malvidin glycosides exhibited greater inhibitory effect than malvidin, whereas Mv-3-glc showed stronger effect than Mv-3-gal in most cases. Mv-3-glc at the concentration of 10 *μ*mol/L reached the greatest inhibition rate. Consistent with ROS results, Mv-3-glc had antagonistic effect with Mv-3-gal at high concentration (10 *μ*mol/L).

**Figure 4:**
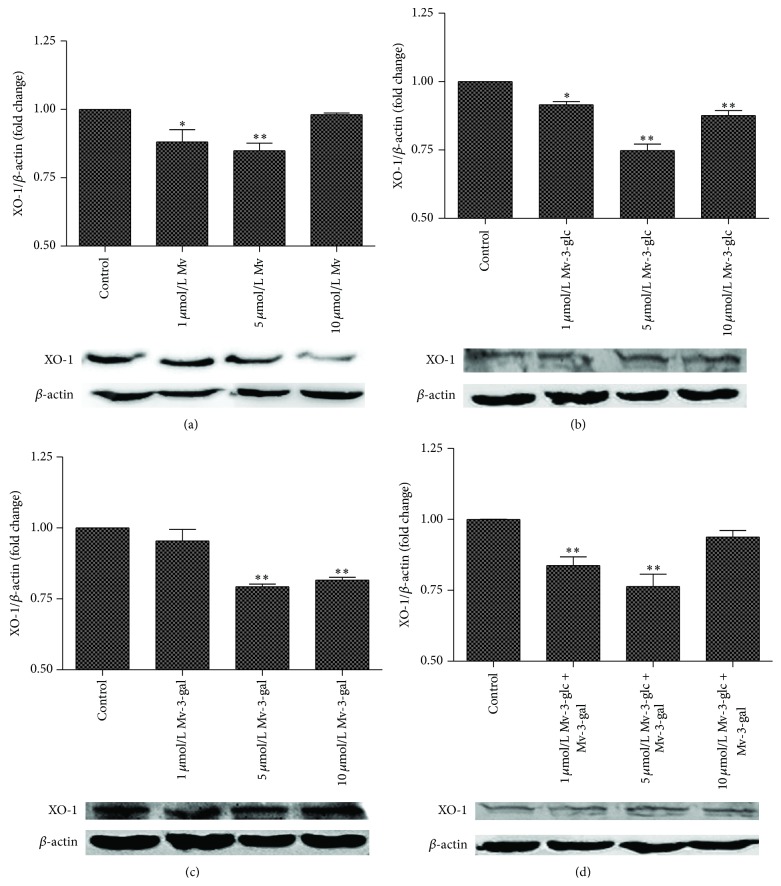
Effects of different concentrations Mv (a), Mv-3-glc (b), Mv-3-gal (c), and Mv-3-glc + Mv-3-gal (d) on XO-1 production in the cells. *∗* and *∗∗* indicate *P* < 0.05 and *P* < 0.01, respectively, compared to the control.

Addition of Mv, Mv-3-glc, Mv-3-gal, and the mixture also decreased the values of XO-1 production in cells to different extents (Figure 4). Low concentration of Mv (1 and 5 *μ*mol/L) had significant inhibitory effects on endothelial XO-1 protein. The relative contents (XO-1/*β*-actin) were 0.88 and 0.86 times compared to those of the control, respectively. The XO-1 relative contents in the cells treated with 1, 5, and 10 *μ*mol/L Mv-3-glc, Mv-3-gal, and the mixture were 0.91, 0.74, 0.87 and 0.95, 0.79, 0.81, and 0.83, 0.76, 0.93 times than those of the control, respectively. High concentration of the mixture (10 *μ*mol/L) had no effect on the XO-1 protein expression in cells, whereas Mv-3-glc tended to produce greater inhibition rate.

### 3.3. Effects of Malvidin and Its Glycosides on SOD Production in Supernatant

**Figure 5:**
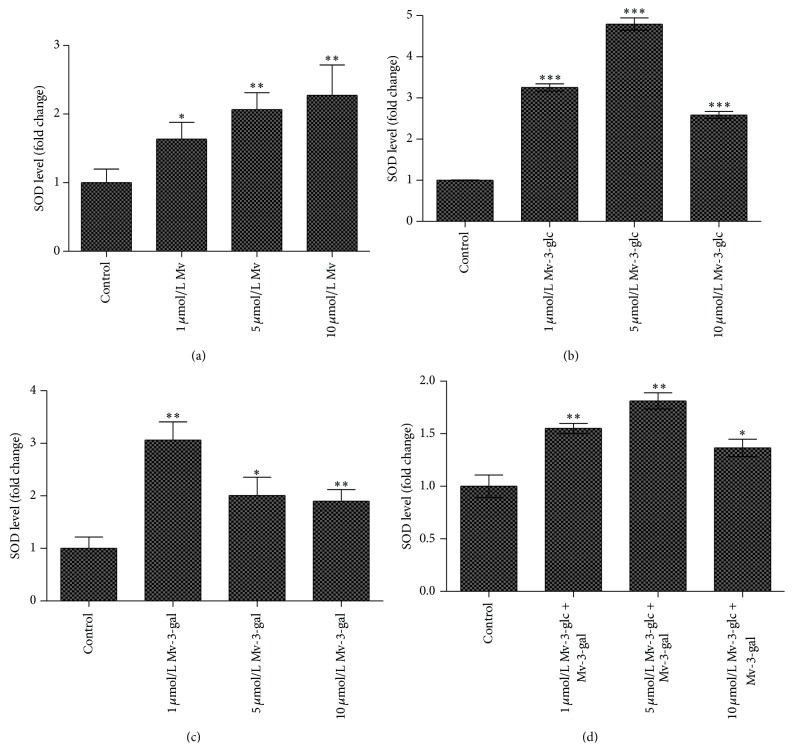
Effects of different concentrations Mv (a), Mv-3-glc (b), Mv-3-gal (c), and Mv-3-glc + Mv-3-gal (d) on SOD level in the supernatant. *∗*, *∗∗*, and *∗∗∗* indicate *P* < 0.05, *P* < 0.01, and *P* < 0.001, respectively, compared to the control.

Malvidin increased the level of SOD production as a function of concentration. When the values of SOD production of the cells treated with 1, 5, and 10 *μ*mol/L malvidin were compared to those of the control, they were found to be 1.64, 2.07, and 2.27 times, respectively. Addition of Mv-3-glc and Mv-3-gal greatly increased SOD production in supernatant. When the SOD production for Mv-3-glc and Mv-3-gal treated at the concentration of 1, 5, and 10 *μ*mol/L was compared to those of the control, they were found to be 3.26, 4.79, 2.59 and 3.06, 2.01, 1.90 times, respectively. However, all products of the mixture of Mv-3-glc and Mv-3-gal at different concentration showed less effect, with changes over the control at 1.55, 1.81, and 1.37 times, respectively (Figure 5).

### 3.4. Effects of Malvidin and Its Glycosides on HO-1 Production in Supernatant

**Figure 6:**
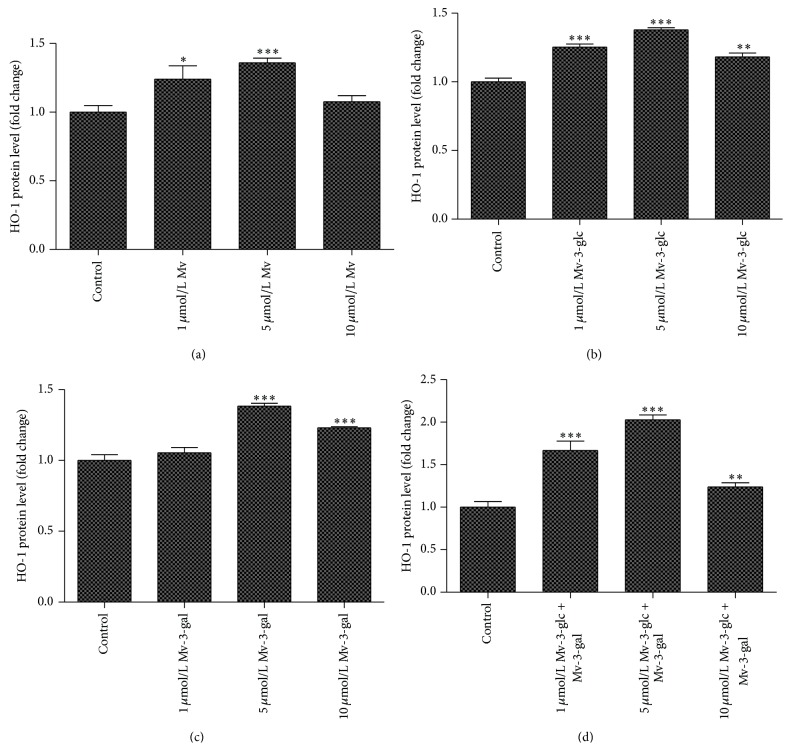
Effects of different concentrations Mv (a), Mv-3-glc (b), Mv-3-gal (c), and Mv-3-glc + Mv-3-gal (d) on HO-1 production released into the supernatant. *∗*, *∗∗*, and *∗∗∗* indicate *P* < 0.05, *P* < 0.01, and *P* < 0.001, respectively, compared to the control.

The control contained 2.09 *μ*g/L HO-1, while the cells treated with 1, 5, and 10 *μ*mol/L malvidin contained 2.59 *μ*g/L, 2.84 *μ*g/L, and 2.24 *μ*g/L HO-1, which were 1.24, 1.36, and 1.08 times than those of the control. The supernatant HO-1 levels of the cells treated with 1, 5, and 10 *μ*mol/L Mv-3-glc, Mv-3-gal, and the mixture were 1.25, 1.38, 1.18, and 1.05, 1.38, 1.23, and 1.67, 2.03, 1.24 times than those of the control, respectively (Figure 6). Addition of malvidin and its glycosides produced a variety of effects on HO-1 production, but they all reached the greatest values at the concentration of 5 *μ*mol/L. There were synergistic effects between Mv-3-glc and Mv-3-gal, since the mixtures at all the concentration showed greater increase rates than the others.

## 4. Discussion

The molecular structure and concentration of anthocyanins in berries determine their bioactivities. Anthocyanins have peculiar chemical structures deficient in electron [23]. The efficacy to scavenge diverse ROS differs from one to another. Generally the antioxidant capacity of anthocyanins is associated with the number of free hydroxyls around the pyrone ring, but this is not always true [24]. Different parameters, including bond dissociation enthalpy, electron transfer enthalpy, electrophilicity, frontier charge density, hardness, ionization potential, and proton affinity, should be calculated to evaluate anthocyanin antioxidant characteristics [7]. A density functional theory study found that antioxidant capacity of different anthocyanin structures was in the following order: cyanidin > malvidin > aurantinidin > delphinidin > peonidin > pelargonidin [25]. Blueberries are known to contain a significant level of anthocyanins, in which malvidin-3-galactoside and malvidin-3-glucoside are the most dominant [6]. Malvidin has four hydroxyls, leading to a good antioxidant capacity. Therefore, blueberries exhibited obvious superiority in the prevention of chronic diseases caused by oxidative damage.

Antioxidants have been shown to delay, inhibit, and prevent the oxidation by interacting with biological systems through many potential mechanisms, such as absorbing oxygen radicals, chelating of the metal ions, scavenging free radicals, regulating enzyme activity and protein levels, and blocking signaling pathways [26]. In the present study, blueberry anthocyanins, malvidin, and its glycosides could reduce oxidative stress and alleviate harmful effects by greatly decreasing the level of ROS in endothelial cells, as well as changing several key proteins' levels. Xanthine oxidase-1 (XO-1), a major source of superoxide, has been implicated in endothelial dysfunction partly due to the rapid inactivation of nitric oxide [27]. Superoxide dismutase (SOD) and heme oxygenase-1 (HO-1) are both the important antioxidant defense against endothelial oxidative damage [28, 29]. The capacity of Mv, Mv-3-glc, Mv-3-gal, and the glycoside mixture on decreasing XO protein level and increasing SOD and HO-1 protein levels further confirmed that blueberry anthocyanins are a potential source of antioxidants.

Generally glycosylation of an anthocyanin seems to decrease the antioxidant capacity compared with the aglycone because it reduces free hydroxyls and metal chelation sites [30]. However, Kähkönen and Heinonen found that different glycosylation patterns either enhanced or diminished the antioxidant power depending on the anthocyanidin and models used for antioxidant analysis because the* in vitro* effect of glycosylation on antioxidant activity depended on the environment when oxidations occurred [31]. Fukumoto and Mazza reported that antioxidant activity increased with hydroxyl groups but decreased with glycosylation of anthocyanidins. Interestingly, this study showed that malvidin-3-glucoside and malvidin-3-galactoside had better antioxidant capacity than malvidin in endothelial cells [32]. In addition, glucoside seemed to be more effective than galactoside on antioxidant improvement. It further confirmed that the molecular structure of glucosylation was the most important factor in determining antioxidant properties of anthocyanins [24]. The results showed that the typical major dietary anthocyanins in blueberries, Mv-3-glc and Mv-3-gal, had great antioxidant properties in endothelial cells. It indicated that they could protect cells from oxidative deterioration and used as a potential functional food ingredients to prevent diseases related to oxidative stress.

## 5. Conclusions

In the present study, treatment with malvidin, malvidin-3-glucoside, malvidin-3-galactoside, and the mixture of the two glycosides significantly attenuated oxidative stress in human umbilical vein endothelial cells. Mv, Mv-3-glc, Mv-3-gal, and the mixture all showed good antioxidant capacity in cells by the mechanism of inhibiting ROS and XO-1 levels and increasing the SOD and HO-1 levels. In most cases, Mv-3-glc had better potential antioxidant effect than Mv-3-gal. This indicated that bioactive anthocyanins in blueberries, such as Mv-3-glc and Mv-3-gal, could be applied in the production of smart and innovative pharmaceutical or functional food ingredients to improve endothelial function and prevent the progression of diseases caused by oxidative stress.
